# The Regulation or the Abolition of Midwives

**Published:** 1898-02-19

**Authors:** 


### The Regulation or the Abolition of Midwives.

Two Bills dealing with the midwives question lie before us, one of which is worthy of careful and serious consideration, while the other, whatever it may be worthy of, will, we fear, only arouse feelings of amusement. The first is the Bill prepared by what is known as the Midwives Bill Committee. It is entitled the Midwives Registration Bill, and was introduced into the House of Commons by the Right Hon. John B. Balfour on the 11th of this month. We understand that the second reading is fixed for May 11th, when the first place on the list for the day has been secured for it. The result of this will be that the Bill cannot be blocked, as it was last year, at the instigation of the British Medical Association, and must come to a division on the second reading. This, then, is a real live Bill, and deserves careful consideration. We cannot but feel that its authors have made a great mistake, just as the authors of the British Medical Association's Bill did, in introducing a definition of the word midwife which does not cover the midwife as she now exists. Endless loopholes are thereby created. In its main provisions, however, the Bill seems well calculated to carry out its purpose of ensuring that those who practice the art of midwifery shall at least have some elementary knowledge of its principles. It forbids the use not only of the name of "midwife," but of any other name, title, or description, implying that the user is specially qualified to act as a midwife unless she be registered; it imposes penalties for breaking this rule; it appoints a Board for regulating the conditions of admission to the register, and for deciding upon the removal from the register of any midwife for misconduct; and it places all midwives under the direct supervision and control of the local sanitary authorities and the medical officers appointed by them as local supervising authorities. The weak part of the Bill is that there is in it no security that the poor will get the cheap and good attendance in labour, the provision of which is the only valid excuse for demanding legislation on the subject, while, on the other hand, there is no security that the class of midwives legalised under this Bill will be restrained from undertaking duties which Parliament has already decided to be the proper work of qualified medical practitioners. All depends on the action of the Board to be appointed under the Act. If this Board should take note of the memorandum which precedes the Bill, and so arrange the education of the midwives as to provide attendance in their confinements to the poorer class of women, who, it is said, "cannot afford even the very lowest fee charged by doctors" then the midwifery of the country will be handed over by law to a very inferior stamp of half-educated women; while if the Board demand anything like a complete knowledge of midwifery, the expense of the education will be such that the fees which will be charged by the new race of midwives will not be appreciably lower than "the very lowest fee charged by doctors," and so the Act will fail of its main purpose. It may be said at once that the Bill will be a failure unless it ensures that the midwives provided by it are both reliable and cheap, and these are two attributes which are not easily combined.

As for the draft of a Sick and Obstetric Nurses Bill, prepared by Dr. Alexander McCook Weir, which has been forwarded to us, it is difficult to speak with becoming gravity. It must be understood that this is not a Bill for the regulation of, but for the abolition of midwives. It has for its object "to abolish the practice of midwifery by ignorant and incompetent persons calling themselves midwives . . . and to make better provision for the nursing of the sick and parturient poor and their children." The Act is to be an adoptive one, but where it is adopted—maybe by District and Parish Councils, or by Voluntary Nursing Associations (!) acting independently of, or in conjunction with, any of the aforesaid authorities, "it shall be unlawful for any person (male or female) to assume the title of midwife, or to practice as such, or to act as a sick or obstetric nurse . . . without the supervision and control of a . . . registered medical practitioner. Any person infringing this provision, and neglecting or refusing to send for medical or surgical aid at or immediately after a confinement, shall be liable to a fine of £10, or imprisonment for a month in default."

All this is, of course, extremely delightful. No doubt the author of the scheme is a very well-meaning person, but surely he can hardly imagine that any moderately sane Parliament would make it "unlawful for any person . . . to act as a sick . . . nurse . . . without the supervision and control of a . . . registered medical practitioner." What are we to think of a man's knowledge of the world when he gravely proposes to inflict fine and imprisonment on "any person" who refuses to send for medical aid at, or immediately after, a confinement? But we need say nothing more.

